# Bidirectional associations between eosinophils, basophils, and lymphocytes with atopic dermatitis: A multivariable Mendelian randomization study

**DOI:** 10.3389/fimmu.2022.1001911

**Published:** 2022-12-09

**Authors:** Zhang Zeng-Yun-Ou, Jian Zhong-Yu, Li Wei

**Affiliations:** ^1^ Department of Dermatology, West China Hospital, Sichuan University, Chengdu, China; ^2^ Department of Urology, Institute of Urology (Laboratory of Reconstructive Urology), West China Hospital, Sichuan University, Chengdu, China; ^3^ Department of Dermatology and Rare Disease Center, West China Hospital, Sichuan University, Chengdu, Sichuan, China

**Keywords:** eosinophils, basophils, lymphocytes, atopic dermatitis (AD), Mendelian randomization

## Abstract

**Background:**

Despite being prone to reverse causation and having unmeasured confounding factors, many clinical observational studies have highlighted the critical association between basophils, eosinophils, and lymphocytes and atopic dermatitis (AD). Whether these cells play a causal role in AD development remains uncertain.

**Methods:**

Data were obtained from the UK Biobank and the Blood Cell Consortium, from a large publicly available genome-wide association study (GWAS) with more than 500,000 subjects of European ancestry and for AD from three independent cohorts with more than 700,000 subjects of European ancestry. We performed single-variable Mendelian randomization (SVMR), followed by multivariable Mendelian randomization (MVMR) to assess the total and direct effects of immune cell counts on AD risk.

**Results:**

SVMR estimates showed that genetically predicted higher eosinophil [odds ratio (OR): 1.23, 95% confidence interval (CI): 1.17–1.29, *p* = 5.85E−16] and basophil counts (OR: 1.11, 95% CI: 1.03–1.19, *p* = 0.004) had an adverse effect on the risk of AD, while a higher lymphocyte count (OR: 0.93, 95% CI: 0.89–0.98, *p* = 0.006) decreased the risk. Reverse MR analysis showed higher basophil (beta: 0.04, 95% CI: 0.01–0.07, *p* = 0.014) and lower lymphocyte counts (beta: −0.05, 95% CI: −0.09 to −0.01, *p* = 0.021) in patients with AD. In MVMR, the effects of eosinophils (OR: 1.19, 95% CI: 1.09–1.29, *p* = 8.98E−05), basophils (OR: 1.19, 95% CI: 1.14–1.24, *p* = 3.72E−15), and lymphocytes (OR: 0.93, 95% CI: 0.89–0.98, *p* = 0.006) were still significant.

**Discussion:**

Mendelian randomization (MR) findings suggest that an increase in the eosinophil and basophil counts and a decrease in the lymphocyte counts are potential causal risk factors for AD. These risk factors are independent of each other.

## Introduction

Atopic dermatitis (AD) is a relapsing chronic inflammatory skin disease characterized by intense itching episodes, elevated eosinophilia, and a dysregulated immune response. The global prevalence of AD is approximately 15%–20% in children and 1%–3% in adults. It has increased two- to threefold, reaching 15%–20% among children and up to 10% among adults, with over 15% overall prevalence in Europe (1, 2). The Global Burden of Disease study showed that AD has the highest disease burden and seriously affects the quality of life and social functioning of patients (3). The complex interplay between immune cells, which leads to immune dysfunction, is an underlying pathogenic mechanisms (4). Many clinical observational studies have revealed that basophils, eosinophils, and lymphocytes are dysregulated in peripheral blood and skin lesions, highlighting their role in the development of AD (5–9).

Basophils are rare circulating granulocytes associated with pruritus that promote AD-like allergic skin inflammation (10). They are activated systemically and promote immune dysregulation in the AD models (10–12). However, the specific mechanism of pathogenesis remains unclear. The activation of eosinophils in peripheral blood and skin lesions is critical for the pathogenesis of AD. Elevated eosinophil levels are risk factors characteristic of AD (13–15) and serve as clinical biomarkers for its assessment. However, the limitations of observational studies result in a lack of evidence highlighting the increased eosinophil count as a definite risk factor. Immune dysregulation in AD is also related to lymphocytes. Typically, the peripheral blood lymphocyte count of patients is normal, and the role of lymphocyte subtypes in immune dysregulation has garnered more interest than the overall lymphocyte count (16, 17). However, the relationship between peripheral blood lymphocyte count and AD is unclear.

Eosinophils, basophils, and lymphocytes play critical roles in augmenting the immune response in AD. Observational epidemiological designs are prone to reverse causation and unmeasured confounding, and the causal role of immune cells in AD development remains uncertain. Mendelian randomization (MR), which is widely used to explore the causality between candidate risk factors and diseases (18, 19), employs single-nucleotide polymorphisms (SNPs) as genetic tools and reliably estimates their effects on the outcomes of interest. The MR approach investigates causal relationships by exploiting genetic variants as instrumental variables (IVs) that influence exposure status, thereby accounting for observational study bias in epidemiological studies, including reverse causation (18). Therefore, we used this method to evaluate the causal relationships between AD and immune cells, specifically the number of eosinophils, basophils, and lymphocytes.

## Materials and methods

### Study design

**Figure 1:**
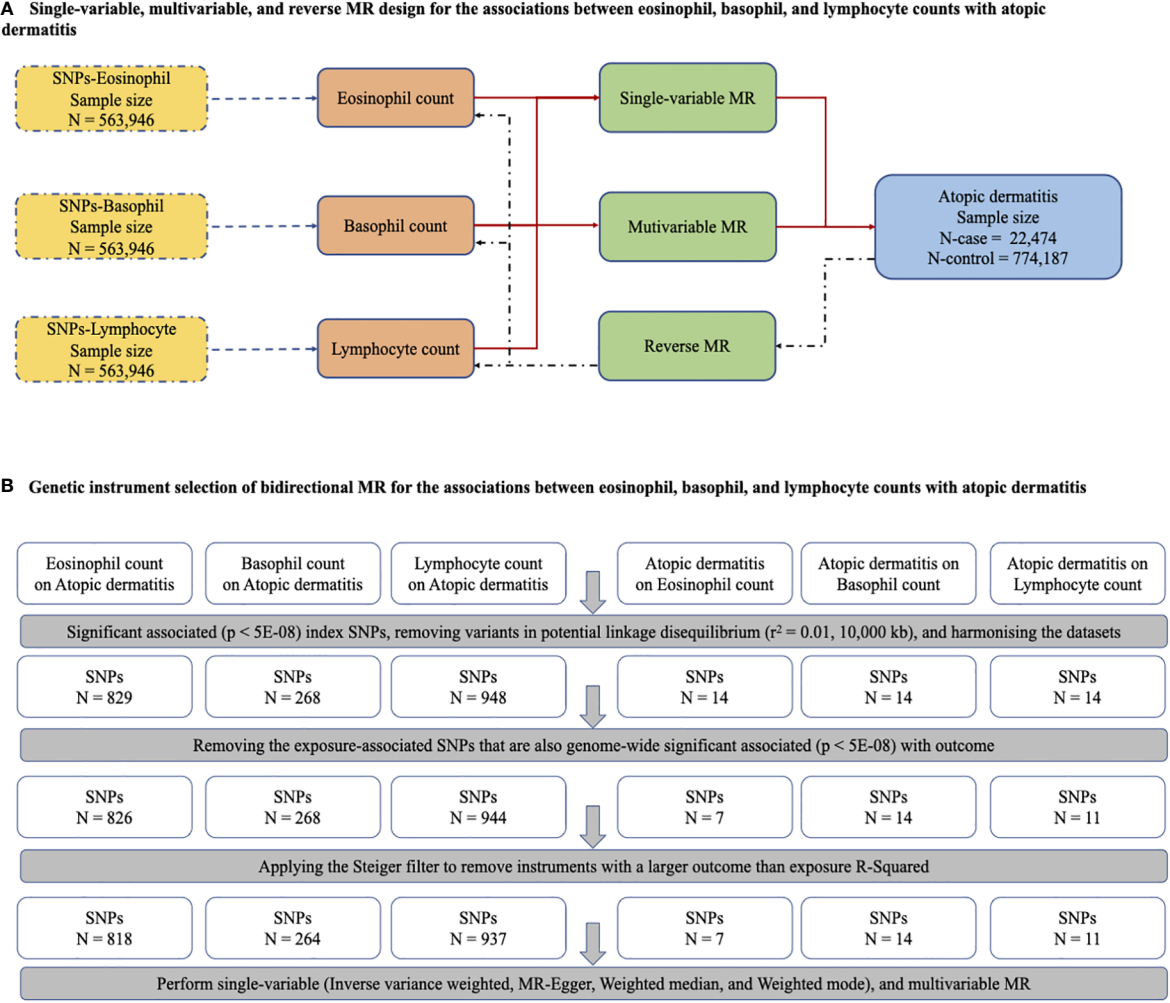
The main design of MR, identification of genetic instruments, and data and MR methods used for analyses. **(A)** Single-variable, multivariable, and reverse MR design for the association between eosinophil, basophil and lymphocyte with atopic dermatitis. **(B)** Genetic instrument selection of bidirectional MR for the associations between eosinophil, basophil and lymphocyte with atopic dermatitis. SNPs, single-nucleotide polymorphisms; MR, Mendelian randomization.

Single-variable MR (SVMR) and reverse MR were conducted to explore the bidirectional associations between immune cell counts (eosinophils, basophils, and lymphocytes) and AD. Given the interplay among the three cell types, multivariable MR (MVMR) was performed to assess the influence of these cell types independently on AD risk (**Figure 1A**). SVMR [inverse variance weighted (IVW), MR–Egger, weighted median, and weighted mode] and MVMR were applied to the estimates. When the genetic variants were independent of each other and no evidence of targeted pleiotropy was observed in the selected IVs (Egger-intercept *p*-value > 0.05), the IVW method was considered the most efficient.

These methods provide useful sensitivity analyses to explore whether the variants are pleiotropic. MR analysis uses IVW as the primary method when all the genetic variants are valid IVs. MR estimates are reported as odds ratios (ORs), which are interpreted as the risk of AD observed with a per-unit increase in inverse normalized immune cell count. The per unit of immune cell count was defined as the relative count (per unit volume of blood) of all immune cells (eosinophils, basophils, and lymphocytes) that was log_10_ transformed before regression modeling and then inverse normalized for cohort level association analysis or genome-wide association studies (GWAS) (20).

### Data resources

A multiple-sample MR analysis with multiple genetic variants as instrumental variables was performed based on summary statistics. Summary statistics of SNPs were retrieved for immune cells (eosinophils, basophils, and lymphocytes) from a subset of the GWAS, which included 29 blood cell phenotypes, to perform a genome-wide discovery analysis examining 563,946 participants of European ancestry from the UK Biobank cohort and the Blood Cell Consortium (21, 22). For AD, we used the summary GWAS statistics, with FinnGen Preparatory Phase Data Freeze 6 comprising data corresponding to 244,544 Finnish adults (8,383 AD cases; 236,161 controls), the Estonian Biobank cohort data from 136,724 participants (11,187 AD cases; 125,537 controls), and the UK Biobank data from 2,904 AD cases and 412,489 controls. The details are presented in **Supplementary Table 1**.

### Instrument selection

All genetic variants that were characterized as significantly associated index SNPs (*p* < 5E−08) with immune cell counts (eosinophils, basophils, and lymphocytes) were selected as IVs. Since the SNPs were in a state of corresponding linkage disequilibrium, we harmonized the datasets by removing variants in potential linkage disequilibrium (*r* ^2^ = 0.01, 10,000 kb). Next, SNPs that were also significantly associated with outcomes (*p* < 5E–08) were removed, and the Steiger filter was applied to eliminate instruments with a larger outcome than the exposure *R* ^2^. Finally, criteria-compliant SNPs were included in our model of bidirectional MR analysis, including the counts on AD of eosinophils (SNPs, *N* = 818), basophils (SNPs, *N* = 264), and lymphocytes (SNPs, *N* = 937), and AD on the counts of eosinophils (SNPs, *N* = 7), basophils (SNPs, *N* = 14), and lymphocytes (SNPs, *N* = 11) (**Figure 1B**). The *F* statistics related to the proportion of variance in the exposure explained by the genetic variants and the strength of all 2,051 SNPs as genetic instruments measured by the *F* statistics ranged from 70.58 to 171.22, indicating a smaller possibility of weak IV bias (**Supplementary Table 2**). The details of all SNPs for immune cells are presented in **Supplementary Table 3**.

### MR assumption

Three key assumptions were made for each of the genetic variants in this MR analysis. First, in the relevance assumption, the genetic variants were associated with the risk factor of interest in a large genome-wide study and the IVs with blood cells with GWAS significance. Next, in the independence assumption, the associations between IVs and outcomes had no unmeasured confounders. Finally, in the exclusion restriction assumption, IVs affected the outcome only through their effect on the risk factor of interest. Genetic variants may affect the outcome through pathways other than the target exposure factor of interest. When genetic variants have pleiotropic effects, IVW, MR–Egger, weighted median, and weighted methods are used to estimate the robust effects. The weighted median method provides reliable evidence when at least half of the valid instrumental variables have no pleiotropic effects, while MR–Egger regression provides consistent estimates when 100% of genetic variants are invalid IVs. In addition, we reduced the weak association between potential confounders and genetic variations by rigorous screening for IVs.

### Statistical analysis

Analyses were performed using the packages Two-Sample MR and MVMR in R v.4.0.3 (www.r-project.org), and power calculations were performed using an online tool (http://cnsgenomics.com/shiny/mRnd/).

## Results

### Univariable MR analysis of the association between eosinophil, basophil, and lymphocyte counts and AD risk

**Figure 2:**
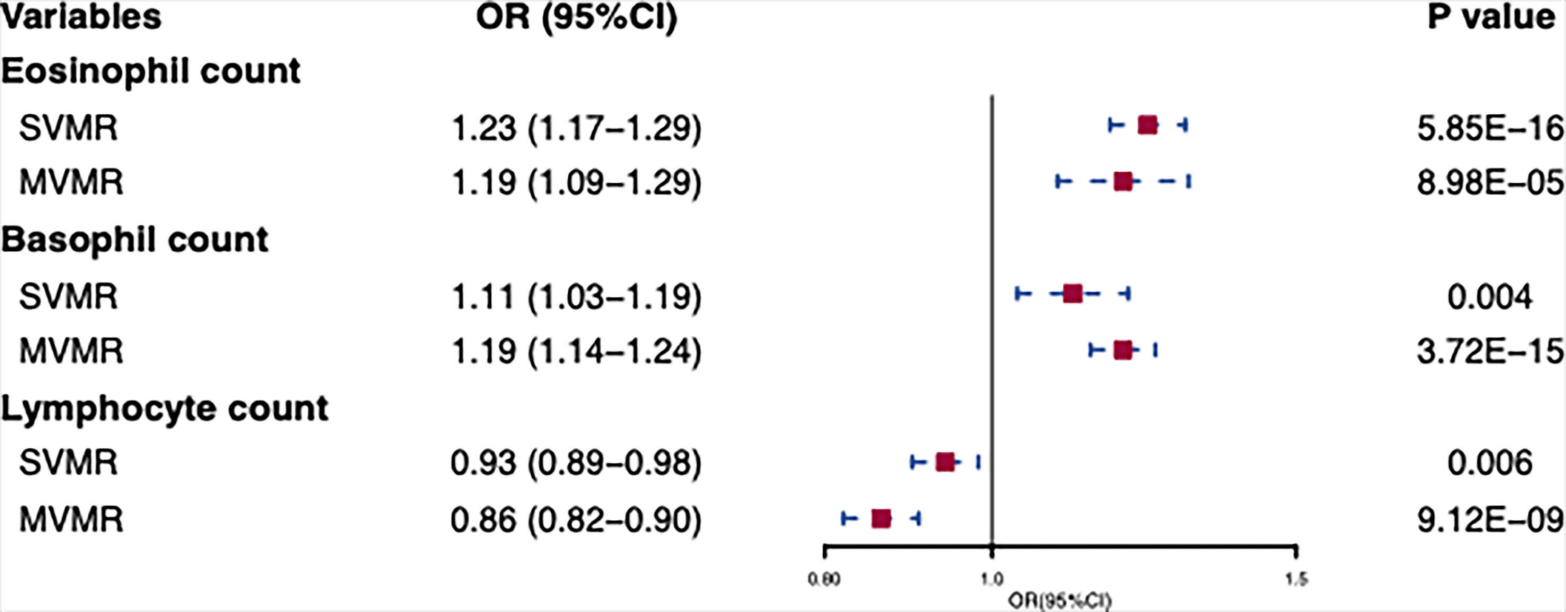
Univariable and multivariable MR of the effect of eosinophils, basophils, and lymphocytes on atpic dermatitis. OR, odds ratio; CI, confidence interval; SVMR, Single-variable MR; MVMR, multivariable MR.

**Figure 3:**
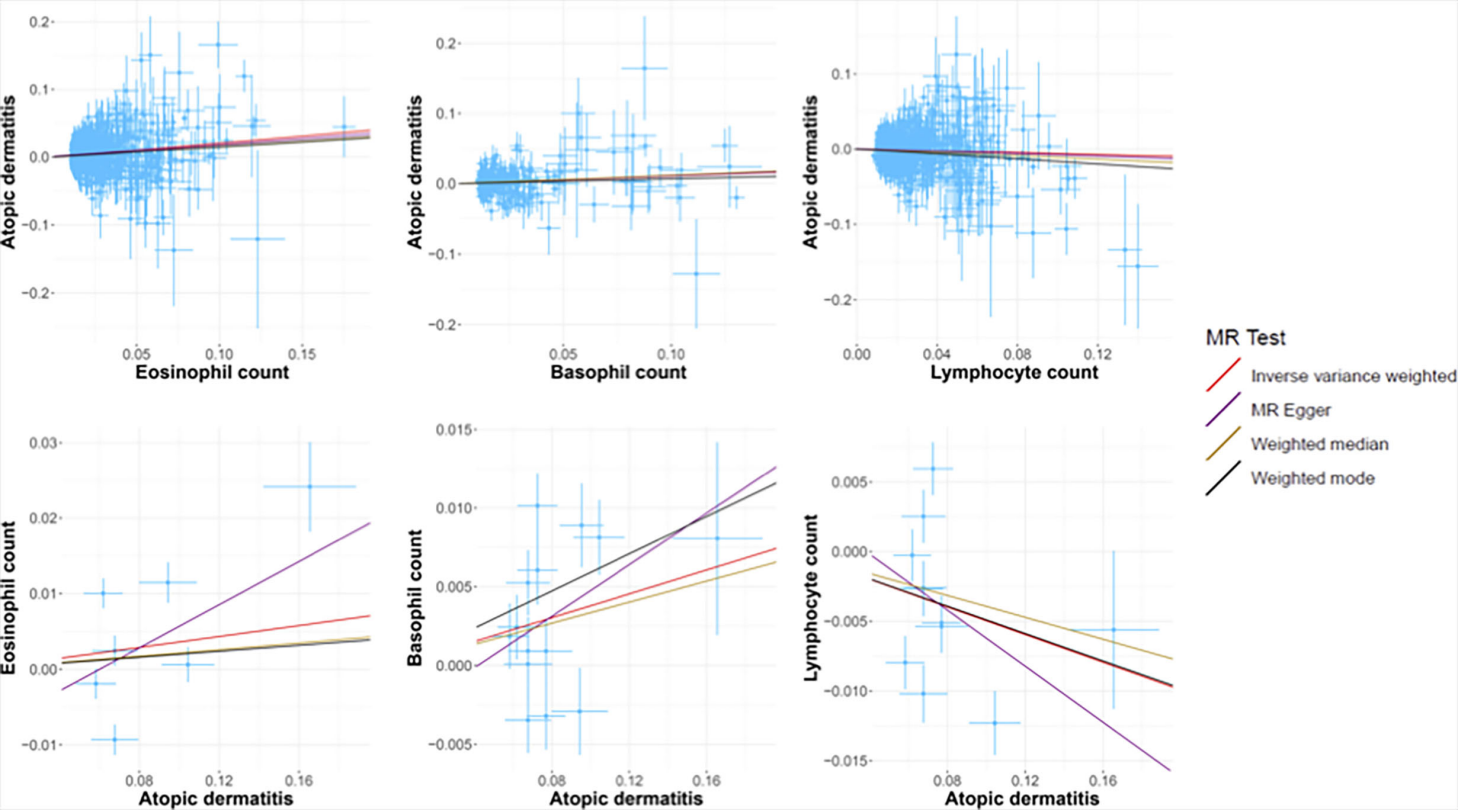
The scatter plots of all MR test results. The red, purple, yellow, and black line represents the IVW, MR-Egger, Weighted median, and Weighted mode effect, respectively. The slope of the line represents the MR effect size.

In standard IVW SVMR analysis, after removing pleiotropic SNPs, we found evidence for genetically predicted eosinophil count (OR: 1.23, 95% CI: 1.17–1.29, *p* = 5.85E−16) with an effect estimate that was consistent with an increased risk for AD (**Figure 2**). The estimates were consistent between MR–Egger sensitivity analyses and the weighted median methods (**Figure 3**). SVMR analyses also showed that the basophil count (OR: 1.11, 95% CI: 1.03–1.19, *p* = 0.004) was associated with an increased risk of AD (**Figure 2**). The sensitivity analysis results showed a consistent trend (**Figure 3**). However, genetic variants associated with lymphocyte count (OR: 0.93, 95% CI: 0.89–0.98, *p* = 0.006) showed a decreased risk for AD (**Figure 2**). The effect directions of the sensitivity analyses were consistent (**Figure 3**). The weighted model (OR: 0.85, 95% CI: 0.75–0.96, *p* = 0.007) showed that a 1-unit increase in lymphocyte count (per nl volume of blood) was causally associated with a 15% relative decrease in AD risk. For all considered outcomes, the intercept test of the Egger-intercept *p*-value did not demonstrate statistical significance and did not indicate horizontal pleiotropy (**Supplementary Table 2**).

We also performed reverse causation analyses. When performing an MR analysis with AD as the exposure, reverse causation indicated a significant effect of the immune cell count on AD (**Table 1**). In our analyses, we observed a strong causal association between AD and basophil (beta: 0.04, 95% CI: 0.01–0.07, *p* = 0.014) and lymphocyte counts (beta: −0.05, 95% CI: −0.09 to −0.01, *p* = 0.021). However, reverse causation analyses between AD and eosinophil (beta: 0.05, 95% CI: −0.04 to −0.11, *p* = 0.354) count did not offer meaningful insights. The scatter plots of all the MR tests also showed a consistent trend (**Figure 3**).

**Table 1:** Associations between eosinophil, basophil, and lymphocyte counts with atopic dermatitis using two-sample Mendelian randomization.

Exposure	Outcome	N-SNPs	MR—method	OR (Beta)	95% LCI	95% UCI	*p*-value	Heterogeneity (*I* ^2^)	Egger-intercept *p*-value
Eosinophil count	Atopic dermatitis	818	IVW	1.23	1.17	1.29	5.85E−16	47%	
			MR Egger	1.19	1.08	1.32	6.43E−04		0.466
			Weighted median	1.17	1.09	1.25	3.87E−06		
			Weighted mode	1.16	0.96	1.41	0.121		
Basophil count	Atopic dermatitis	264	IVW	1.11	1.03	1.20	0.004	24%	
			MR Egger	1.12	0.97	1.29	0.125		0.965
			Weighted median	1.13	1.02	1.25	0.025		
			Weighted mode	1.07	0.87	1.32	0.516		
Lymphocyte count	Atopic dermatitis	937	IVW	0.94	0.90	0.98	0.006	28%	
			MR Egger	0.92	0.84	1.01	0.065		0.571
			Weighted median	0.89	0.83	0.96	0.001		
			Weighted mode	0.85	0.75	0.96	0.007		
Atopic dermatitis	Eosinophil count	7	IVW	0.04	−0.04	0.11	0.354	92%	
			MR Egger	0.14	−0.15	0.44	0.388		0.496
			Weighted median	0.02	−0.02	0.06	0.271		
			Weighted mode	0.02	−0.03	0.06	0.420		
Atopic dermatitis	Basophil count	14	IVW	0.04	0.01	0.07	0.014	73%	
			MR Egger	0.08	−0.07	0.24	0.321		0.580698484
			Weighted median	0.03	0.01	0.06	0.014		
			Weighted mode	0.06	−0.01	0.13	0.131		
Atopic dermatitis	Lymphocyte count	11	IVW	−0.05	−0.09	−0.01	0.021	86%	
			MR Egger	−0.10	−0.32	0.12	0.387		0.650469385
			Weighted median	−0.04	−0.07	−0.01	0.009		
			Weighted mode	−0.05	−0.10	0.00	0.076		

### Multivariable MR analysis of the association between eosinophil, basophil, and lymphocyte counts and AD risk

In IVW-MVMR, upon assessing the association of genetic liabilities between immune cell count and AD, eosinophil (OR: 1.19, 95% CI: 1.09–1.29, *p* = 8.98E−05) and basophil counts (OR: 1.19, 95% CI: 1.14–1.24, *p* = 3.72E−15) retained a positive relationship with AD (**Figure 2**), while lymphocyte count (OR: 0.93, 95% CI: 0.89–0.98, *p* = 0.006) had a negative relationship (**Figure 2**). These results were broadly consistent with those observed in SVMR. In MVMR–Egger sensitivity analyses, horizontal pleiotropy was not indicated, leading to very similar effect estimates (eosinophil count, OR: 1.15, 95% CI: 1.09–1.22, *p* = 9.77E−07; basophil count, OR: 1.19, 95% CI: 1.09–1.30, *p* = 1.2E−04; lymphocyte count, OR: 0.86, 95% CI: 0.82–0.90, *p* = 3.48E–09) to those observed with IVW-MVMR.

## Discussion

Using conventional MVMR, potential bidirectional associations between the genetic liability for immune cell count and AD were evaluated, and genetic evidence indicated that an increase in the count of eosinophils and basophils was associated with increased AD risk. Strikingly, a decrease in lymphocyte count increased AD risk in individuals of European descent.

Immune cell count estimates were consistent in magnitude and direction across IVW, weighted median, weighted mode, and MR–Egger analyses. Furthermore, as typically expected in MR genetic association studies, eosinophil and basophil counts indicated a strong positive association with AD, but lymphocyte count had the opposite relationship. The results of MVMR analysis were consistent with those of SVMR analyses. The MR–Egger and Egger-intercept *p*-value results in the MR analysis indicated the absence of pleiotropy. These findings validated the clinical phenomenon reported in observational literature that indicates an increase in some immune cells in the peripheral blood of patients with AD (14, 23), revealing that increased eosinophil and basophil counts increased AD risk. A decrease in lymphocyte count could increase the risk of AD; however, this hypothesis has garnered little interest in clinical settings.

In atopic diseases, eosinophils are recruited and activated by thymic stromal lymphopoietin and interleukin 33 (IL-33). Activated eosinophils migrate to the target organ and enhance Th2 responses by inducing the expression of specific chemokines such as CCL22 and CCL17 (24). Many studies have observed that eosinophils are typically increased in the peripheral blood in atopic diseases (AD, asthma, allergic rhinitis, eosinophilic esophagitis, and food allergy) (13, 23, 25). Although atopic disorders differ in their pathogenesis, they share a similar atopic phenotype, such as an increased eosinophil count in the peripheral blood. Many observational studies have shown that personal or parental history of allergic rhinitis and asthma are risk factors for the development of AD (26–28). Compared to the general population, patients with other atopic diseases have a higher rate of AD (29). This may be related to the activation and increased number of eosinophils in the blood. However, this hypothesis cannot be proven because of the complex pathogenesis of atopic disorders and many confounding factors. Since univariable MR analyses indicate eosinophil count to be a strong causal risk factor for AD but do not support a causal relationship between AD and eosinophil count, our findings provide genetic evidence that eosinophil count is a risk factor for AD.

Although basophils constitute only <1% of peripheral blood leukocytes in humans, they play a critical role in mediating immune mechanisms and are promising novel markers for measuring the severity of AD. Basophils are associated with the development of inflammation and barrier function imbalance in AD (11, 12) and are activated by immunoglobulin (Ig)E. They may also be activated independently of IgE. Activated basophils are increased in skin lesions and peripheral blood and promote inflammation and itching by secreting histamine, Th2 cytokines (basophil-derived IL-4 promotes Th2 differentiation), proteases, and eicosanoids (30, 31). Two observational studies found a positive association between AD and basophil count in peripheral blood. Although peripheral basophil count was significantly higher in patients with AD than in healthy controls (23, 32), it has not been proven to be a risk factor for AD. In this study, we found genetic evidence that increased basophil count was associated with increased AD risk and *vice versa*. These findings imply that patients with AD may have a higher peripheral blood basophil count that may not be above the normal upper limit compared to that in healthy individuals. Individuals with high basophil levels are at a higher risk of AD than healthy individuals. This may explain why the basophil count in the blood of patients with AD may be normal.

B and T lymphocyte imbalance is an important immune mechanism involved in AD. Recently, the specific subtypes of lymphocytes and their roles, rather than their overall count, have garnered significant interest. The findings of two retrospective studies indicating that the peripheral lymphocyte count of the AD group is higher than that of the healthy controls (33, 34) need to be confirmed by epidemiological data and clinical studies. SVMR analysis proved the negative relationship between lymphocyte count and AD and *vice versa*. A 1-unit increase in lymphocyte count was causally associated with a 15% relative decrease in AD risk. This was the first study to propose a relationship between the lymphocyte count and AD. Although the lymphocyte count is normal in the blood of most patients with AD, lower lymphocyte counts may be detrimental to disease control. Our findings indicate that the normal ranges of lymphocyte count in the blood may not be appropriate for patients with AD.

Redefining the range of these cells in specific disorders may be beneficial for the prevention and surveillance of AD. Disorders characterized by increased eosinophils and basophils or decreased lymphocytes in the peripheral blood should be examined, as they may also induce AD. Overall, our findings fill the gap in the association between the number of eosinophils, basophils, and lymphocytes and AD observed in previous epidemiological observational studies.

### Strength and limitations

This study had several strengths. The large sample size in GWAS increased the measurement precision. The MVMR models had major strengths, and rigorous screening of IVs greatly improved the confidence of our results. The consistent trends observed in the MR sensitivity analysis indicated high reliability. The limitations of this study should also be noted. Although the sensitivity analyses failed to find evidence of horizontal pleiotropy, we could not exclude the association, which could be mediated *via* other causal pathways. Next, as our cohorts included mostly European populations, caution is warranted before applying the findings to non-European populations. Finally, the sourcing of our cohorts from different countries in Europe may have resulted in data overlap; however, the inclusion of larger cohorts may reduce this effect.

## Conclusion

MR findings suggest that an increase in the eosinophil and basophil counts and a decrease in the lymphocyte counts are potential causal risk factors for AD. These risk factors are independent of each other.

## Data availability statement

The datasets presented in this study can be found in online repositories. The names of the repository/repositories and accession number(s) can be found below: https://www.ncbi.nlm.nih.gov/genbank/, The GWAS data of immune cells were from MRC Integrative Epidemiology Unit (IEU) OpenGwas project (ieu-b-33 for eosinophils, ieu-b-29 for basophils and ieu-b-32 for lymphocytes) https://www.ebi.ac.uk/metagenomics/, The GWAS data of AD were from the NHGRI-EBI Catalog (https://www.ebi.ac.uk/gwas/) with ID GCST90027161.

## Author contributions

LW and JZ-Y conceived the study, participated in its design and coordination, and critically revised the manuscript. JZ-Y and ZZ-Y-O searched the databases, and reviewed the GWAS datasets and finished the data collection. JZ-Y finished the data analysis. ZZ-Y-O drafted the manuscript. All authors contributed to the article and approved the submitted version.
