# Supplementary material for: Bidirectional associations between eosinophils, basophils, and lymphocytes with atopic dermatitis: A multivariable Mendelian randomization study
Source: Front Immunol. 2022 Dec 9;13:1001911. doi: 10.3389/fimmu.2022.1001911 (PMC9780468; doi:10.3389/fimmu.2022.1001911)
Supplement: Supplementary file 1 [file Table_1.docx]

Supplementary table 1. The details of GWAS data information for eosinophils, basophils, lymphocytes and AD

| Exposures | Consortium | First Author (Year) | Sample Size in UK Biobank | | Units | Description | MRC-IEU id: | Population | PMID |
| --- | --- | --- | --- | --- | --- | --- | --- | --- | --- |
| Eosinophil count | Blood Cell Consortium | Vuckovic (2020) | 563,946 | 408,112 | SD | Count of eosinophils per unit volume of blood | ieu-b-33 | Mixed | 32888494 |
| Basophil count | Blood Cell Consortium | Vuckovic (2020) | 563,946 | 408,112 | SD | Count of basophils per unit volume of blood | ieu-b-29 | European | 32888494 |
| Lymphocyte count | Blood Cell Consortium | Vuckovic (2020) | 563,946 | 408,112 | SD | Aggregate count of lymphoid cells per unit volume of blood | ieu-b-32 | European | 32888494 |
| Atopic dermatitis | NA | Sliz (2022) | 796661 (22,474 cases) | 415,393  (2,904 cases) | logOR | Atopic dermatitis in FinnGen, Estonian Biobank, and UK Biobank | NA | European | 34454985 |
