# Supplementary material for: Bidirectional associations between eosinophils, basophils, and lymphocytes with atopic dermatitis: A multivariable Mendelian randomization study
Source: Front Immunol. 2022 Dec 9;13:1001911. doi: 10.3389/fimmu.2022.1001911 (PMC9780468; doi:10.3389/fimmu.2022.1001911)
Supplement: Supplementary file 2 [file Table_2.docx]

Supplementary table 2. The calculated power of MR study

| Variables | Samplesize (Exposure) | Samplesize (Outcome) | nSNP | Proportion of variance explained | F statistics | Type-I error rate | Calculated power |
| --- | --- | --- | --- | --- | --- | --- | --- |
| Eosinophil count on Atopic dermatitis | 563,946 | 796,661 (22,474 cases) | 818 | 10.2% | 78 | 0.05 | 100% |
| Basophil count on Atopic dermatitis | 563,946 | 796,661 (22,474 cases) | 264 | 3.2% | 71 | 0.05 | 85% |
| Lymphocyte count on Atopic dermatitis | 563,946 | 796,661 (22,474 cases) | 937 | 10.8% | 73 | 0.05 | 85% |
| Atopic dermatitis on Eosinophil count | 796,661 (22,474 cases) | 563,946 | 7 | 0.1% | 114 | 0.05 | NA* |
| Atopic dermatitis on Basophil count | 796,661 (22,474 cases) | 563,946 | 14 | 0.3% | 171 | 0.05 | NA* |
| Atopic dermatitis on Lymphocyte count | 796,661 (22,474 cases) | 563,946 | 11 | 0.2% | 145 | 0.05 | NA* |

R2, the proportion of variance in the exposure explained by the genetic variants, was calculated using the TwoSampleMR R functions get_r_from_pn for continuous traits and get_r_from_lor for binary traits.

The F statistics related to the proportion of variance in the exposure explained by the genetic variants(R2), samplesize(N) and number of instruments(K) and was calculated by the formula F = ((N-K-1)/K ) ( R2/(1-R2)).

The power calculation was performed with an online tool (<http://cnsgenomics.com/shiny/mRnd/>).

*We did not calculate the power because the exposure is binary traits.
